# Supplementary material for: Polyketide synthase gene clusters in the ericoid mycorrhizal fungus Oidiodendron maius: genome-wide prediction, expression profiling, and functional characterization of knockout mutants
Source: Front Plant Sci. 2026 Sep 16;17:1924430. doi: 10.3389/fpls.2026.1924430 (PMC13623903; doi:10.3389/fpls.2026.1924430)
Supplement: Supplementary file 1 [file SupplementaryFile1.zip › Supplementary Materials/Supplementary Folder 1.zip!/Oidma1_AssemblyScaffolds_Repeatmasked/knownclusterblast/cluster13/jgi.p_Oidma1_25908_mibig_hits.html]

| MIBiG Protein | Description | MIBiG Cluster | MiBiG Product | % ID | % Coverage | BLAST Score | E-value |
| --- | --- | --- | --- | --- | --- | --- | --- |
| CAE02620.1 | Yx01\_protein | BGC0000433 | Surfactin\_biosynthetic\_gene\_cluster | 24 | 73.2240437158 | 74 | 2.2e-13 |
| CAF32372.1 | putative\_3-amino-2,3-dideoxy-scyllo-inositol\_1-dehydrogenase | BGC0000712 | Paromomycin\_biosynthetic\_gene\_cluster | 23 | 88.5245901639 | 72 | 8.5e-13 |
| BAK64639.1 | putative\_dehydrogenase | BGC0000135 | Reveromycin\_biosynthetic\_gene\_cluster | 24 | 92.8961748634 | 62 | 8.8e-10 |
| BAE95593.1 | putative\_dehydrogenase | BGC0000703 | Kanamycin\_biosynthetic\_gene\_cluster | 26 | 48.3606557377 | 61 | 2.6e-09 |
| CAF60509.1 | putative\_dehydrogenase | BGC0000704 | Kanamycin\_biosynthetic\_gene\_cluster | 26 | 48.3606557377 | 61 | 2.6e-09 |
| CBW45668.1 | putative\_zinc-containing\_alcohol\_dehydrogenase\_superfamily\_protein | BGC0000952 | Pristinamycin\_biosynthetic\_gene\_cluster | 21 | 91.5300546448 | 51 | 2.6e-06 |
| CAE45671.1 | borrelidin\_polyketide\_synthase,\_type\_I | BGC0000031 | Borrelidin\_biosynthetic\_gene\_cluster | 31 | 24.8633879781 | 50 | 4.5e-06 |
